# Transition of Treatment for Ground Glass Opacity–Dominant Non-Small Cell Lung Cancer

**DOI:** 10.3389/fonc.2021.655651

**Published:** 2021-04-15

**Authors:** Yoshinori Handa, Yasuhiro Tsutani, Morihito Okada

**Affiliations:** Department of Surgical Oncology, Hiroshima University, Hiroshima, Japan

**Keywords:** non-small cell lung cancer, ground glass opacity (GGO), lobectomy, sublobar resection (SLR), prognosis

## Abstract

Lobectomy has been the standard surgical treatment for non-small cell lung cancer (NSCLC). Over the decades, with the dramatic development of radiographic tools, such as high-resolution computed tomography (HRCT), and the widespread practice of low-dose helical CT for screening, the number of cases diagnosed with small-cell lung cancers with ground glass opacity (GGO) at early stages has been increasing. Accordingly, mainly after 2000, many retrospective studies and prospective trials have shown that patients with lung adenocarcinoma with GGO have a good prognosis and may be candidates for sublobar resection. Previous studies indicated that HRCT findings including the maximum diameter of the tumor, GGO ratio, and a consolidation/tumor ratio (CTR) are simple and useful tools to predict tumor invasiveness and prognosis in patients with NSCLC with GGO. Thus, sublobar resection may be considered a “standard therapy” for peripheral GGO-dominant small-cell lung adenocarcinomas. Ultimately, some of such tumors might not require surgical resection. A multicenter, prospective study has just begun in Japan to evaluate the validity of follow-up for small-sized GGO-dominant small-cell lung cancer. Lung cancers that do not require surgery should be identified. This study reviewed retrospective and prospective studies on GGO tumors and discussed the treatment strategies for such tumors.

## Introduction

Lobectomy has been the standard surgical treatment for non-small cell lung cancer (NSCLC), even when it is in its early stage. In 1973, Jensik et al. (1) suggested that segmental resection is equivalent to lobectomy and represent an adequate operation for small stage I NSCLC. This article started a debate regarding the optimal surgical approach for early-stage NSCLC. In 1995, however, a randomized trial reported that sublobar resection for stage IA NSCLC did not result in improved morbidity, mortality, or postoperative pulmonary function and was associated with higher rates of locoregional recurrence and death relative to lobectomy (2). In this trial, in patients who underwent sublobar resection, recurrence showed a 75% increase (p = 0.02) attributable to a tripling of the local recurrence rate (p = 0.008), 30% increase in overall death rate (p = 0.08), and 50% increase in death due to cancer (p = 0.09) compared to patients undergoing lobectomy. Two years later, another multicenter study showed a similar trend in increased local recurrence in patients who underwent sublobar resection (wedge resection) (3). Eventually, sublobar resection has been performed only for patients unable to tolerate lobectomy as a “somewhat poor quality” alternative.

Over the decades, with the dramatic development of radiographic tools, such as high-resolution computed tomography (HRCT) and the widespread practice of low-dose helical CT for screening, lung cancers, especially many small lung cancers with ground glass opacity (GGO), are increasingly diagnosed. Recently, approximately 40% patients who underwent operations were reported to be diagnosed with stage IA NSCLC (4). Therefore, treatment of patients with very early-stage lung cancer *via* lobectomy, which is a more aggressive procedure than sublobar resection, has become controversial. In the 2000s, many retrospective studies have demonstrated that patients with lung adenocarcinoma with GGO have good prognoses and the potential to be candidates for sublobar resection. Now, appropriate treatment for NSCLC with GGO needs to be fully discussed.

We reviewed the literature and summarized the “new trend in early-stage lung cancer presenting as GGO,” mainly in terms of surgical treatment. Thus, we evaluated appropriate treatment strategy for NSCLC with GGO.

## Prognosis of NSCLC With GGO Component and Sublobar Resection

Mainly after 2000, several clinical studies have demonstrated that patients with lung adenocarcinoma with GGO have good prognoses (5–8). We examined 436 of 502 consecutive patients with stage IA adenocarcinoma and had undergone preoperative HRCT; 66 patients with tumors with pure GGO were excluded. Tumor type (without GGO, n = 137; with GGO, n = 299) and surgical results were analyzed. Tumors without GGO showed a significantly greater association (P < 0.001) with lymphatic, vascular, and pleural invasion and lymph node metastasis compared with tumors with GGO. Namely, most tumors with GGO are diagnosed as pathological non- or less-invasive lung adenocarcinomas, such as adenocarcinoma *in situ* (AIS), minimally invasive adenocarcinoma (MIA), former bronchioloalveolar carcinoma, and Noguchi type A-B adenocarcinomas, which presented similar results to those of a previous report (5). Additionally, Asamura et al. previously analyzed the correlation between radiologic findings of GGO-dominant tumors and pathological characteristics and reported that GGO lesions constitute true early lung cancers, namely, minimal or noninvasive tumors (9). The disease-free survival also worsened in patients with pure solid tumors (P = 0.0006) (4). Similarly, Hattori et al. retrospectively evaluated 1029 surgically resected early-stage NSCLCs. All tumors were classified into two groups: with GGO group or pure solid group. They revealed that on multivariable analysis, the presence of a GGO was an independent significant prognostic factor of overall survival (OS) (hazard ratio [HR], 0.314; 95% confidence interval [CI], 0.181–0.529: P < 0.001) (6). In particular, GGO-dominant lung cancer was reported to have excellent prognosis. Asamura et al. (5) reported that in the Japan Clinical Oncology Group [JCOG] 0201 study, patients diagnosed with GGO-dominant lung adenocarcinoma have a good prognosis (**Table 1**). This prospective, multi-institutional study was performed with 233 male and 312 female patients (median age, 62 years) to define early (noninvasive) adenocarcinomas of the lung on image diagnosis,; the median follow-up period of all patients was 7.1 years (range, 0–8.5 years). The JCOG 0201 study showed that the 5-year OS was 96.7% for patients with a consolidation/tumor ratio (CTR) ≤ 0.5 and a ≤30 mm tumor, and 97.1% for those with CTR ≤ 0.25 and a ≤20 mm tumor. In addition, the incidence of pathological invasiveness (pathological N+ or ly+ or v+) was 1.1% in patients with a CTR ≤ 0.5 and a ≤30 mm tumor, and 0.3% in those with CTR ≤ 0.25 and a ≤20 mm tumor. This study concluded that the radiologic criteria of a CTR ≤ 0.25 and ≤20 mm, and CTR ≤ 0.50 in ≤30 mm were both able to define a homogeneous group of patients with an excellent prognosis before surgery. In addition, we demonstrated that patients with GGO-dominant (CTR ≤ 0.5) lung adenocarcinomas rarely had pathologically invasive tumors and had an excellent prognosis (8) (**Table 1**). We evaluated 610 consecutive patients with clinical stage IA lung adenocarcinoma who underwent complete resection after preoperative HRCT and revealed 239 (39.2%) patients were CTR ≤ 0.5. Based on the results, no significant difference in 3-year recurrence-free survival (RFS) was observed among patients who underwent lobectomy (96.4%), segmentectomy (96.1%), and wedge resection (98.7%) of GGO-dominant tumors (P = 0.44). Multivariate Cox analysis showed that surgical procedure did not affect RFS in GGO-dominant tumors. We revealed that GGO-dominant clinical stage IA lung adenocarcinomas are a uniform group of tumors that exhibit low-grade malignancy and have an extremely favorable prognosis and thus can be successfully treated with sublobar resection. In particular, in a ≤20 mm tumor, surgeons can successfully treat lung cancer patients *via* wedge resection that does not include lymph node dissection. The prognosis of patients with lung adenocarcinoma is better when tumors include GGO, compared with those with pure solid tumors. However, whether the prognosis of patients with tumors with GGO tumors is favorable regardless of the solid component size remains unknown. We retrospectively analyzed the clinicopathological findings and prognoses of 856 patients with tumors with GGO based on the size of the solid component during a median follow-up of 45 months; among the 1215 patients with lung adenocarcinoma, it was revealed that the prognostic impact of a solid component size less than or equal to 2 cm and >2 cm significantly differed after complete resection (10).

**Table 1:** Summary of previous large cohort studies evaluating GGO dominant NSCLC.

	*n*	Follow up	Size	CTR	Performed Surgical Procedure WR/Sg/Lob	Pathologically invasiveness (pN+ or ly+ or v+)	Prognosis
JCOG 0201 (7)	35	7.1y	≤20mm	≤0.25	0(0%)/0(0%)/35(100.0%)	1 patient (2.9%)	**5-year OS**; 97.1% **5-year RFS**; 97.1%
	54	7.1y	21–30 mm	≤0.5	0(0%)/0(0%)/54(100.0%)	N.S	**5-year OS**; 96.3% **5-year RFS**; 94.4%
	121	7.1y	≤30mm	≤0.5	0(0%)/0(0%)/121(100.0%)	6 patients (5.0%) (breakdown N.S)	**5-year OS**; 96.7% **5-year RFS**; 95.9%
JCOG 0804 (10)	314	5.5y	≤20mm	≤0.25	258(77.5%)/56(22.5%)/0(0%)	N.S	**5-year RFS**; 99·7%
Tsutani et al. (8)	239	3.5y	≤30mm	≤0.5	93(38.9%)/56(23.4%)/90(37.7%)	3 patients with ly+ (1.3%) 2 patients with v+ (0.8%) 1 patient with pl+ (0.4%) 2 patients with pN+ (0.8%)	**3-year OS** 98.7%(WR)/98.2%(Seg)/97.6%(Lob)**3-year RFS** 98.7%(WR)/96.1%(Seg)/96.4%(Lob)

CTR, consolidation/tumor ratio; Lob, lobectomy; N.S, not stated; OS, overall survival; RFS, recurrence free survival; Seg, segmentectomy; WR, wedge resection.

**Figure 1:**
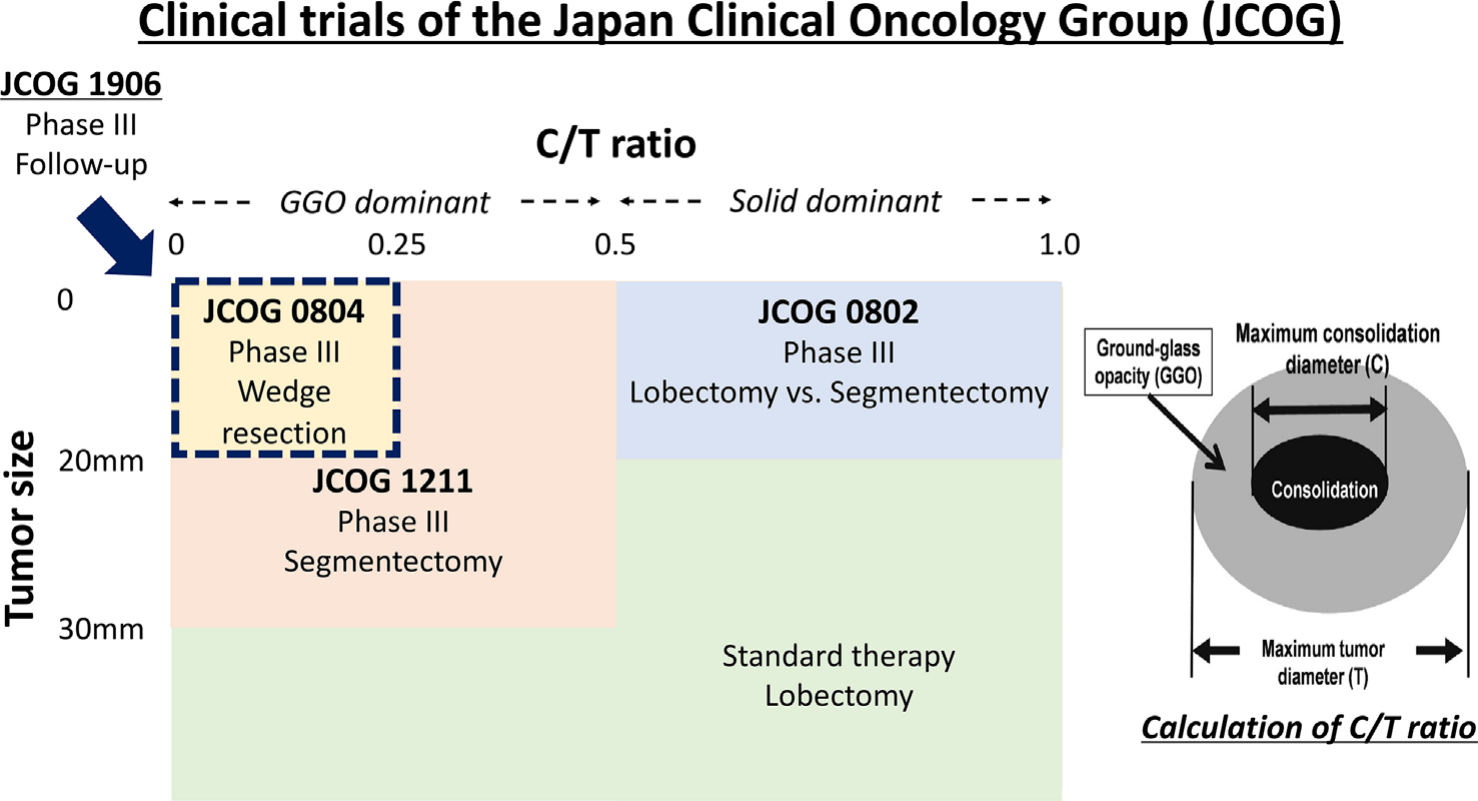
Schema of clinical trials of the Japan Clinical Oncology Group (JCOG) Study.

Preoperative HRCT findings (GGO ratio, CTR) showed that GGO is a simple and useful tool for predicting the prognosis of NSCLC candidates for sublobar resection. The nonrandomized confirmatory phase III study (JCOG 0804/WJOG 4507L) conducted by the JCOG and West Japan Oncology Group (WJOG) evaluated the efficacy and safety of sublobar resection for GGO-dominant lung cancer defined with HRCT only by prospective, multicenter, and large cohort analysis (314 registered patients) (**Figure 1**). The selection criteria were maximum tumor diameter ≤ 20 mm, CTR ≤ 0.25, no recurrence of the original lung cancer, and a 5-year RFS rate of 99.7% after sublober resection, primarily wedge resection (**Table 1**). Their results were clearer and showed that HRCT was very useful in selecting patients suitable for sublobar resection (11).

Another prognostic factor used as a surrogate of tumor aggressiveness is the high tumor maximum standard uptake value (SUVmax) on 18F-fluorodeoxyglucose-positron emission tomography/CT (FDG-PET) (12–16). High SUV max NSCLC demonstrate invasive pathologic characteristics, and poorer postoperative outcomes were suggested when compared with tumors with lower SUV max. Previous study reported that a higher ratio of tumor SUVmax to tumor size was associated with worse DFS (12). Additionally, hypermetabolic tumors were reported to be associated with substantially higher invasiveness (13). Other studies also have reported that a higher tumor SUVmax was associated with higher grade tumors and aggressive histopathologic subtypes (i.e., micropapillary and solid subtypes of adenocarcinoma), and in many cases, NSCLC with GGO have lower SUV max tumor on FDG-PET (14). We have also observed that SUVmax on FDG-PET was an important preoperative factor for predicting the pathologic malignant grade and prognosis in lung adenocarcinoma (15, 16). As stated in our paper (8), GGO-dominant tumors have a low median SUVmax of 0.9 and are generally low in malignancy, but some of the highest tumors have a SUVmax of 9.8. Furthermore, GGO-dominant tumors showing a high SUVmax may be pathologically malignant and have lymph node metastasis. We previously identified the predictors of pathologic lymph node involvement in clinical stage IA lung adenocarcinoma (17). In this study, we revealed the pathologic node-negative status criteria of solid tumor size <0.8 cm on HRCT or a SUVmax <1.5 on FDG-PET/CT. Therefore, conversely, in other cases, lymph node metastasis cannot be denied. In combination with other pieces of literature (12–16), tumors showing a high SUVmax need to be resected by more than segmentectomy, even when it is constituted by the GGO component. We think that the treatment strategy is clearly different depending on SUVmax; namely, for small GGO-dominant tumors with low standard uptake value (SUV), it may be reasonable to be resected by wedge resection, but for those with high SUV, more than segmentectomy should be considered. The role of PET/CT might be limited in the evaluation of very small lung nodules, however, the good prognosis of NSCLC with GGO can be confirmed from the viewpoint of FDG-PET.

Sublobar resection has several advantages than lobectomy. It reportedly improves postoperative quality of life by preserving the pulmonary function (18, 19). Harada et al. reported that a positive and significant correlation was found between the number of resected segments versus loss of forced vital capacity (r = 0.518, p < 0.0001 at 2 months; r = 0.604, p < 0.0001 at 6 months) and loss of forced expiratory volume in 1 second (r = 0.492, p < 0.0001 at 2 months; r = 0.512, p < 0.0001 at 6 months). The postoperative reduction of forced vital capacity (p = 0.0006) and forced expiratory volume in 1 second (p = 0.0007) was significantly less in the segmentectomy group. They concluded that the extent of removed lung parenchyma directly affected that of postoperative functional loss even at 6 months after surgery, and segmentectomy offered significantly better functional preservation compared with lobectomy (18). We emphasized that preserving as much healthy lung tissue as possible reduces the prevalence of surgery and improves postoperative quality of life. In addition, patients with GGO-dominant lung cancer survive long enough to be at risk for the next lung cancer, increasing the likelihood of further resection (19). The smaller the initial amount of excision, the more unlimited treatment options for subsequent lung cancer. Perhaps, some surgeons may be concerned that sublobar resection may increase local recurrence, however, in combination with these circumstances, it may be reasonable to perform sublobar resection as the “standard treatment” for lung adenocarcinoma with a maximum tumor diameter of 20 mm or less and a CTR of 0.25 or less.

## Expansion of Indications for Segmentectomy in 21–30 mm NSCLC With GGO Component

Sublobular resection is generally considered lung cancer smaller than 20 mm. However, the excellent prognosis of GGO-dominant lung cancer allowed us to consider expanding its indications for sublobar resection. JCOG 0201 shows that patients with a CTR of 0.5 or less and tumors of 21-30 mm have a 5-year OS of 96.3% and a 5-year RFS of 94.4% (5). In addition, GGO-dominated tumors of 21-30 mm rarely showed pathological invasiveness, and there was no difference in survival analysis, specifically, for tumors of 21-30 mm, where GGO predominates, 3-year RFS was similar in patients who underwent lobectomy (93.7%) and segmentectomy (92.9%). Therefore, we have shown that GGO-dominant and 21-30 mm tumors may also be candidates for sublobar resection (8). It was necessary to distinguish between wedge resection and segmentectomy to clarify which procedure was used. We recommended segmentectomy and not wedge resection for sublobar resection of 21–30 mm tumors because these tumors could metastasize to lymph node, and taking a sufficient surgical margin often is difficult in a 21–30 mm tumor. In our study, 2 (2.4%) of 84 patients with a GGO-dominant 21-30 mm tumor metastasized to the lymph nodes, but in patients with a GGO-dominant tumor of 20 mm or less, no lymph node metastasis was seen. Among sublobar resections, segmentectomy involves anatomical resection in the hilar region, thereby allowing more lymph nodes to be dissected. The prognostic impact of lymph node dissection on lung cancer treatment has remained unclear. The main role of lymph node dissection has been to prevent understaging. A higher number of lymph nodes sampled during surgery improve the accuracy of pathologic staging, thereby preventing the misclassification of patients with lymph node involvement as having stage I disease (20). Moreover, in several reports, it has been shown that lymph node dissection has an important prognostic impact. In previous studies using SEER data, it has been shown that the number of lymph nodes evaluated during surgery was a strong predictor of survival for stage I NSCLC (21). As previously mentioned, we revealed the pathologic node-negative status criteria of solid tumor size of <0.8 cm on HRCT or a SUVmax of <1.5 on FDG-PET/CT (17). Therefore, conversely, lymph node metastasis cannot be denied, and lymphadenectomy should be performed in other cases. The optimal extent of resection margin and lymph node dissection in segmentectomy has not been elucidated, and future study is warranted; however, we believe that segmentectomy is superior to wedge resection when the hilar lymph nodes are dissected and have sufficient surgical margin.

Currently, a nonrandomized confirmatory trial of segmentectomy (JCOG 1211) is underway since September 2013 with the aim of confirming the effectiveness of segmentectomy for clinical T1N0 GGO-dominant lung cancer based on HRCT (**Figure 1**) (22). A total of 390 patients from 42 Japanese institutions are recruited within 4 years. The primary endpoint of this study is a 5-year relapse-free survival in all of the patients who undergo a segmentectomy for a lung nodule. The secondary endpoints are OS, annual relapse-free survival, disease-free survival, proportion of local relapse, postoperative pulmonary function, proportion of segmentectomy completion, proportion of R0 resection completion by segmentectomy, adverse events, and serious adverse events. Patient accrual have already ended in November 2015 and a primary analysis will be conducted in 2021. This study is a crucial trial of lung segmentectomy for early stage lung cancer.

## Gene Expression of NSCLC With GGO Component

Suda K conducted a retrospective analysis of the Japanese Joint Committee of Lung Cancer Registry database (a nationwide database for patients with surgically resected lung cancer; n = 18,973). They evaluated 5780 patients had been tested for an EGFR mutation, and revealed the presence of an EGFR mutation was significantly correlated with the presence of GGO (P < 0.001) and better prognosis (23). GGO component of NSCLC is often pathologically reflect adenocarcinoma *in situ* (AIS), minimally invasive adenocarcinoma (MIA), and lepidic component. We previously evaluated gene expression of NSCLC comprehensively. Ito M et al. evaluated typical driver mutation for lung cancer, EGFR mutation, in 394 resected pN0M0 lung adenocarcinomas (24). In this study, we revealed that the frequency of EGFR mutation is higher in adenocarcinoma with a concomitant lepidic component, such as AIS and MIA. On the contrary, EGFR wild type tumors are likely to be invasive adenocarcinoma cases without a lepidic component. These results revealed that NSCLC with GGO component might have room for therapeutic intervention in terms of gene expression, considering the effectiveness of treatment by epidermal growth factor receptor-tyrosine kinase inhibitors. Further investigations on gene expression in NSCLC with GGO component will be needed.

## Potential of Follow-Up for GGO-Dominant NSCLC

Some small lung cancers with GGO have been reported to have no pathological invasiveness and significantly longer doubling times than normal lung adenocarcinomas (25, 26). Aoki et al. reported that all type A and B tumors by Noguchi criteria had a tumor doubling time of more than 1 year, on the other hand, the tumor doubling time was less than 1 year in almost (87%) of the types D, E, and F tumors. In addition, Kakinuma et al. evaluate the natural course of the progression of pulmonary subsolid nodules (SSNs) (27). A total of 795 patients with 1229 SSNs were included from eight Japanese facilities. SSNs were classified into three categories: pure ground glass nodules (PGGNs), heterogeneous GGNs (HGGNs) (solid component detected only in lung windows), and part-solid nodules. Among the 1046 PGGNs, 13 (1.2%) developed into HGGNs and 56 (5.4%) developed into part-solid nodules. Among the 81 HGGNs, 16 (19.8%) developed into part-solid nodules. For the PGGNs, the mean period until their development into part-solid nodules was 3.8 ± 2.0 years, whereas the mean period for the HGGNs was 2.1 ± 2.3 years (p = 0.0004). In patients who underwent surgical resection, invasive adenocarcinomas were diagnosed only among the part-solid nodules, corresponding to 1% (12 patients) of all 1229 SSNs.

Some GGO-dominant NSCLC might not require surgical resection itself. In several pieces of literature, it has been indicated that the outcomes of stereotactic body radiotherapy and radiofrequency ablation for operable early-stage NSCLC were as good as those in previous surgery studies (28–31). In the future, surgeons may need clinical trials that compare sublobar resection and radiotherapy. In addition, now, multicenter, prospective study has just begun in Japan to evaluate the validity of follow-up for GGO-dominant small lung cancer (maximum tumor diameter ≤2 cm and CTR ≤ 0.25) without pulmonary resection. (JCOG 1906, UMIN000040818) (**Figure 1**). A total of 680 patients from 42 Japanese institutions are planned to be recruited within 5 years. The primary endpoint of this study is a 10-year OS in all of the patients who undergo follow-up. Sublobar resection, even a wedge resection, is a surgical procedure that requires general anesthesia and carries a considerable risk of adverse events. If the surgery itself can be avoided, it is the least invasive treatment for the patient. Ultimately, lung cancer that does not require surgery needs to be identified in the future.

## Discussion

From many retrospective studies and JCOG 0804 trial previously mentioned, we believe that it is rational to perform sublobar resection as the “standard treatment” for lung adenocarcinoma with a maximum tumor diameter of ≤20 mm and CTR ≤ 0.25 (**Figure 1**), and in selecting either segmentectomy or wedge resection, segmentectomy is superior to wedge resection in dissecting the hilar lymph nodes. For lung adenocarcinoma with a maximum tumor diameter of ≤20 mm and 0.25 ≤ CTR ≤ 0.5, the standard is a lobectomy, but sublobar resection is also possible based on past data. If you can get a sufficient surgical margin, wedge resection might be enough. For the treatment of GGO-dominant lung adenocarcinoma with a maximum tumor diameter of 20–30 mm, the standard is a lobectomy, but sublobar resection is also possible based on data as previously described. To secure a surgical margin, surgeons should choose segmentectomy in principle. If surgeons can get sufficient surgical margin, wedge resection can be chosen. Finally, the results of JCOG 1211 must be awaited. Ultimately, lung cancer that does not require lung resection may be identified. Thus, further investigations into the treatment of GGO-dominant lung cancer are needed.

## Author Contributions

YH and YT designed this study. MO supervised the study. YH and YT collected clinical information, and interpreted all of the data. All authors contributed to the article and approved the submitted version.

## Conflict of Interest

The authors declare that the research was conducted in the absence of any commercial or financial relationships that could be construed as a potential conflict of interest.
